# The Organotypic Longitudinal Spinal Cord Slice Culture for Stem Cell Study

**DOI:** 10.1155/2015/471216

**Published:** 2015-01-31

**Authors:** Joanna Sypecka, Sylwia Koniusz, Maria Kawalec, Anna Sarnowska

**Affiliations:** ^1^NeuroRepair Department, Mossakowski Medical Research Centre, Polish Academy of Sciences, 5 Pawinskiego Street, 02-106 Warsaw, Poland; ^2^Molecular Biology Unit, Mossakowski Medical Research Centre, Polish Academy of Sciences, 5 Pawinskiego Street, 02-106 Warsaw, Poland; ^3^Translative Platform for Regenerative Medicine, Mossakowski Medical Research Centre, Polish Academy of Sciences, 5 Pawinskiego Street, 02-106 Warsaw, Poland; ^4^Stem Cell Bioengineering Laboratory, Mossakowski Medical Research Centre, Polish Academy of Sciences, 5 Pawinskiego Street, 02-106 Warsaw, Poland

## Abstract

The objective of this paper is to describe in detail the method of organotypic longitudinal spinal cord slice culture and the scientific basis for its potential utility. The technique is based on the interface method, which was described previously and thereafter was modified in our laboratory. The most important advantage of the presented model is the preservation of the intrinsic spinal cord fiber tract and the ventrodorsal polarity of the spinal cord. All the processes occurring during axonal growth, regeneration, synapse formation, and myelination could be visualized while being cultured *in vitro* for up to 4-5 weeks after the slices had been isolated. Both pups and adult animals can undergo the same, equally efficient procedures when going by the protocol in question. The urgent need for an appropriate *in vitro* model for spinal cord regeneration results from a greater number of clinical trials concerning regenerative medicine in the spinal cord injury and from still insufficient knowledge of the molecular mechanisms involved in the neuroreparative processes. The detailed method of organotypic longitudinal spinal cord slice culture is accompanied by examples of its application to studying biological processes to which both the CNS inhabiting and grafted cells are subjected.

## 1. Introduction

Cell therapy is now considered a new tool to effectively deal with acute or chronic spinal cord injury [1, 2]. Since pathological processes like lesion, demyelination, or inflammation are not followed by spontaneous regeneration of axons in the mature CNS, in the last couple of years a number of applicable strategies have been devised to improve axon repair [3] and to elaborate spinal cord injury treatment [4–7]. More than 23 clinical trials have been open for stem cell therapy dedicated to cure spinal cord injury ([8], http://www.clinicaltrials.gov/). Stem cells delivered to the site of injury are supposed to provide growth factors, cytokines, and other immunomodulatory factors to enhance axonal growth, to reduce inflammation, to boost angiogenesis, and also to rebuild the injured part of the cord [9]. Although the mechanisms of beneficiary effects of stem cell therapies can largely be predicted and a spectrum of tools are available for controlling cell differentiation, a number of experimental studies should still be done simultaneously to optimize the methods of transplantation and to elucidate the mechanisms in action and the therapeutic and the side effects of stem cell therapy. On one hand, there is very limited number of described results coming from studies based on cell culture to look deeply into axonal regeneration processes, while on the other hand the necessity of applying the methods which could frequently be repeated at the early stages of the study precludes the use of animal models. Considering validity of those premises, the organotypic cultures seem to be the optimal method that allows live observation of transplanted cells, significant number of experiment repetitions, and reduction in the number of animals used in research work.

Organotypic slice cultures were established as a model sharing the properties of both cell culture and animal model. Organotypic slices, compared to cell (neurons, astrocytes, and oligodendrocytes) cocultures retain tissue organization and maintain cell-to-cell contact and therefore are more similar to the* in vivo* environment [10]. The slice cultures derived from hippocampus are used most frequently as a model of nervous tissue with the preserved cytoarchitectural organization. However, depending on a pathology-stricken brain segment, also cerebellum [11], forebrain [12], and striatum [13], slice cultures had been established in various laboratories. The abovementioned models allow investigating disorders resulting from different brain disorders like ischemia [14], trauma [15], or toxic injury [16].

To study spinal cord pathology or reparative mechanisms, the experiments should preferably be looked upon in the context of the spinal cord microenvironment [6, 17–19]. As we proved before [20, 21] either the spinal cord or the brain environment exerts a markedly different influence on cultured cells. In order to prove this, transverse organotypic spinal slice cultures were established [22, 23]. The model enables relatively easy visualization of nerve fiber growth, synaptic activity, or network interface using techniques of immunofluorescence, as well as that of scanning and transmission electronic microscopy. The transverse slices could be, however, obtained from only a single part of the spinal cord. In such a model, the longitudinal growth of axons, a quintessential process in many diseases of the spinal cord, was hard to spot. The longitudinal slices enabled to study the mechanisms controlling the process of reinnervation or the proper conduct of axon regrowth [24]. Therefore we have established a technique for preparing a coculture of longitudinal spinal cord slices with stem/progenitor cells as the* in vitro* model for studying cell therapy aimed at spinal cord regeneration. The key advantage of longitudinal* in vitro* slice cultures is the preserved architecture of the intact spinal cord with their long axonal projections. In the described method, the slices are obtained from two-three consecutive spinal cord segments and therefore it is possible to observe the intrinsic spinal cord axons forming a fiber tract. When using the model, the fate of axonal fibers in the presence of various factors or stem cells could be closely followed.

In the last decade a number cell-based treatments have been recommended. They are supposed to stimulate axon regrowth [25, 26] and to prevent the inflammation resulting in glial scar formation [27, 28]. In order to demonstrate that longitudinal spinal cord organotypic slices can actually be cultured* in vitro*, either human umbilical cord derived neural stem cells (HUCB),the line obtained in our laboratory [29], or oligodendroglia progenitors derived from neonatal brains (OPC) [30] were transplanted in two paradigms: directly on top of the spinal cord slice cultures (SCC) or indirectly, that is, below the SCC, so the cells were cocultivated while being separated from SCC by a thin layer of culture medium.

We would like to detail the method of establishing the longitudinal spinal cord organotypic slice culture as the preferable model for elaborating strategies for the cell therapy dedicated to spinal cord injury and additionally to bring out the differences in stem/progenitor cell fate depending on brain or spinal cord microenvironment.

## 2. Materials and Methods

### 2.1. Longitudinal Spinal Cord Slice Culture

**Figure 1:**
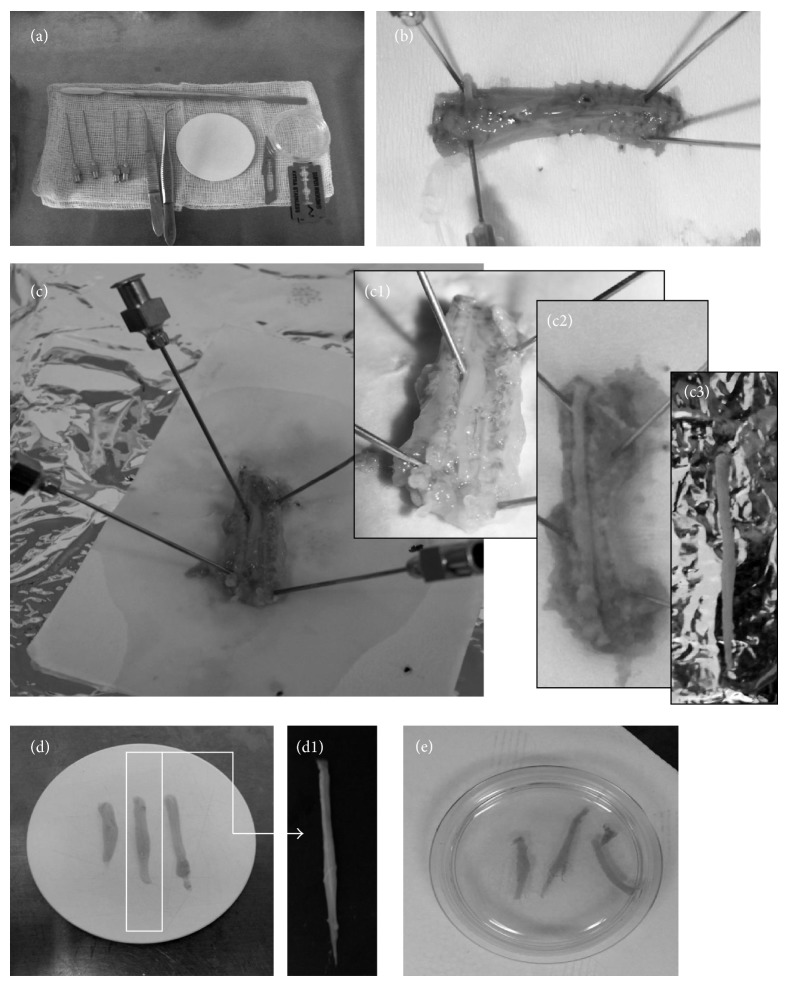
Illustration of sequential steps of organotypic spinal cord slices preparation. All needed for preparation tools (a) and the main steps of the spinal cord isolation were photographed. The incision of the entire spinal cord block from the dorsal side (b). Fixation with syringe needles spinal column (c). Progressive steps of spinal cord isolation (c1–3). The dissection of white spinal cords (d) and its magnification (d1). The transfer of spinal cord slices into the Millicell-CM (Millipore) membranes (e).

The technique is based on the interface method, which was described previously [11] and thereafter was modified in our laboratory [31]. Spinal cord slices were routinely extracted from 5–7-day old Wistar rats. In another experimental variant, the slices were prepared from spinal cords of adult rats (8-week old, 180–200 g weight); however, the procedure was less effective (details in results) and the viability of the culture was significantly poorer. The isolation procedure described above was the same regardless of the age of the animals. After anesthetizing them briefly with Vetbutal (pentobarbital; Sigma), the ice-cooled animals were plunged into 70% alcohol solution and decapitated with scissors, and then the trunk was quickly removed and put on the operating table. The next step was to incise the entire spinal cord block from the dorsal side (Figures 1(a) and 1(b)). Then the spine was cut from the ventral side with a scalpel blade. The spinal column was cut open and the limbs were fixed to the table with syringe needles. In the middle of the exposed backbone, the white spinal cord could be seen (Figure 1(c)). Using a scalpel blade, the spinal roots were gently incised and the spinal cord was dissected (Figure 1(d)). The spine was removed with two metal microspatulas and it was immersed in ice-cold HBSS (Gibco) (Figure 1(e)). To obtain the slices, the spine was placed on the McIlwain tissue chopper, alongside the blade, and was cut into 400 *μ*m slices and 1.5–2-cm-long slices. The slices were transferred onto the Millicell-CM (Millipore) membranes for further growth, three-four slices on each. The Millicell-CM membranes in 6-well plates were preequilibrated with 1 mL of culture medium (pH 7.2, 50% DMEM, 10 mM HEPES, 25% HBSS, 25% horse serum (Gibco), 2 mmol/L L-glutamine, 5 mg/mL glucose, 1% amphotericine B, and 0.4% penicillin-streptomycin) prepared according to Aitken et al. and modified by Gähwiler et al. [32, 33]. The cultures started to grow in a regular, 25% horse serum medium to be gradually replaced (from DIV 4th until 7th) by SF, defined-solution-based medium. The SF medium contained DMEM/F12, 10 mM HEPES, 25% HBSS, 2 mmol/L L-glutamine, 5 mg/mL glucose, 1% amphotericin B and 0.4% penicillin-streptomycin, N2A (1 : 10; Gibco), and the B27 (1 : 100; Gibco) supplements. The cultures were grown in humid conditions and 5% CO_2_, at 36°C for 4-5 weeks.

### 2.2. Isolation of Oligodendroglial Progenitors

Oligodendrocyte progenitor cells were isolated from a mixed glial primary cell cultures established from the neonatal rat brains according to the method previously described in detail [30]. Briefly, the brain hemispheres of Wistar rats Cmd:(WI) WU were mechanically but gently homogenized in Hank's Buffered Salt Solution (Invitrogen, Carlsbad, CA) warmed up to 37°C and filtered through 41 *μ*m Hydrophilic Nylon Net Filter (Millipore, Bedford, MA) in order to eliminate tissue debris. The procedure of animal handling and brain isolation was approved by the IV Local Ethics Committee on Animal Care and Use (Ministry of Science and Higher Education). The obtained single-cell suspension was spun down (1500 g, 10 min), plated into 75-cm^2^ culture flasks (NUNC, Naperville, IL), and cultured for the following 10–12 days (37°C, 5% CO_2_, 95% humidity) in Dulbecco's medium (high glucose) (Gibco) supplemented with 10% fetal bovine serum and penicillin-streptomycin solution (Sigma). Then the mixed primary glial culture served for separation of the fractions of each glial cell types by placing the culture flasks on the orbital shaker SSM1 (Stuart) in the cell incubator. Firstly, the microglial fraction was separated during 1-hour shaking (180 rpm). Then, after replacing the culture medium with the fresh portion, the OPCs were gently detached by shaking the cultures for the following 15–18 h. The floating cells were spun down (1500 g, 10 min), dispersed, and seeded at 2 × 10^5^/cm^2^ density on poly-L-lysine-coated 6-well plates (NUNC). Obtained OPC population was left to adhere in serum-free F12/DMEM medium (Gibco) and then was used for setting cocultures with organotypic spinal cord slices.

### 2.3. Cell Transplantation onto the Top of SCC

**Figure 2:**
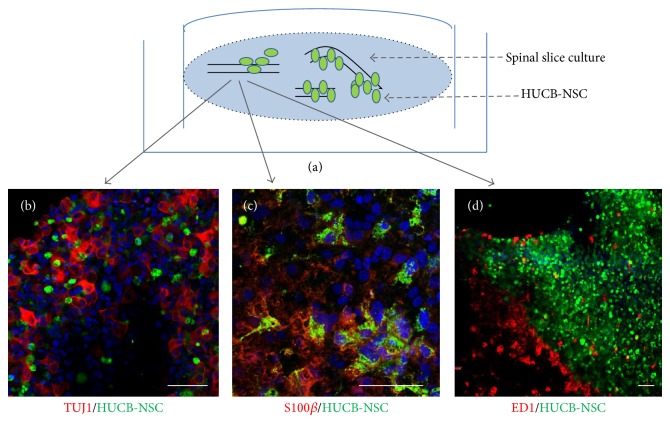
Direct transplantation of HUCB-NSC into the spinal cord organotypic slice culture, an example of immunohistochemistry experiment. The cells were traced with CMFDA for their identification after engraftment. The transplanted cells (green) migrated inside the slice and spread out between the host neurons ((b); TUJ1, red). Three weeks after transplantation part of the transplanted cells expressed the astrocyte marker S100*β* ((c); red). The local immune response to xenografts was moderate ((d); ED1, red). Cell nuclei (blue) were stained with Hoechst 33258. Scale bar is 50 *μ*m.

HUCB-NSCs were transplanted as soon as SCC had been prepared. Approximately 1 × 10^5^ CMFDA-traced HUCB-NSCs were suspended in 20 *μ*L of medium. Then they were spread over the whole slice-covered surface with a pipette. After being transplanted for a day, the cells that failed to stick to the slices were washed out by gently washing membranes with PBS using pipette. Then the slices were cultivated at 36°C for up to one week at 36°C in air + 5% CO_2_ atmosphere of 100% humidity with the medium changed every two days (Figure 2(a)).

### 2.4. Indirect Coculturing of SCC with Stem/Progenitor Cells

**Figure 3:**
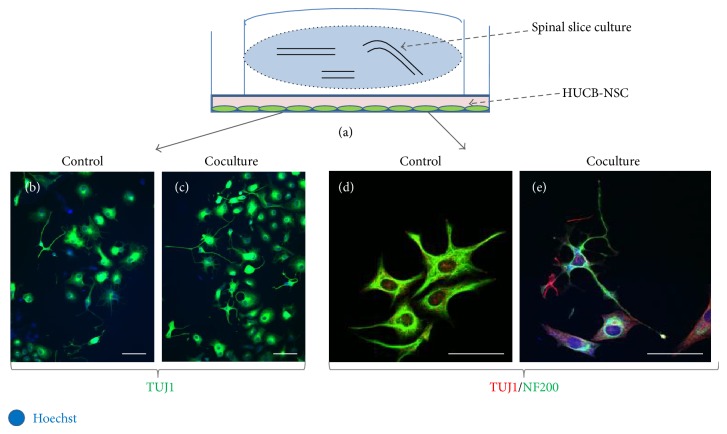
Cocultivation of HUCB-NSC with the spinal cord organotypic slice culture, an example of immunohistochemistry experiment. The cells growing in the 6-well plates were cocultivated with SCC placed on the membranes inserted into wells (a), without a direct cell-to-cell contact with the slices. HUCB-NSC seeded at the beginning of the experiment at the same density proliferated faster in the vicinity of SCC ((b)-(c)). Moreover, their morphology was more ramified and the observed TUJ1 expression was higher than that in the control ((d)-(e)). Cell nuclei (blue) were visualized by staining with Hoechst 33258. Scale bar corresponds to 50 *μ*m.

The spinal cord organotypic slice cultures were transferred into 6-well plates containing on-glass cultured either HUCB-NSC or OPCs. During the following 2 weeks, SCC and the cells were cultivated in the same wells; however, a thin layer of culture medium separated the cells from the slices (Figure 3(a)).

### 2.5. Immunocytochemistry

The spinal cord slices were fixed in 4% paraformaldehyde (PFA) dissolved in PBS for 1 hour. To do this, 1 mL of 4% PFA was poured below the membrane and another 1 mL of 4% PFA was gently poured on top of the slices, respectively. After 1 hour the slices were washed in PBS three times (1 mL below and 1 mL on the top of the membrane) and then shifted from the membrane with a tiny brush to 24-well culture plates filled with PBS. During the next stage the slices were permeabilized with 1% Triton X-100 (Sigma) for 15 min and blocked with a PBS-diluted 5% NGS. The blocking media were applied for 1 hour at room temperature. Primary antibodies were diluted as follows: monoclonal antibodies anti-TUJ1 (IgG2a, 1 : 1000; Covance), anti-*β*-tubulin III (1 : 500, Sigma), NF200 (IgG1, 1 : 400; Sigma), and ED1 (IgG1, 1 : 100; Serotec), and then they were incubated overnight at 4°C. Polyclonal antibodies either anti-GFAP (1 : 200; DAKO) or anti-S100*β* (1 : 1000; Swant) were also applied overnight at 4°C. The proliferating cells were stained with monoclonal antibody against Ki67 (1 : 100; Novocastra). As a control, the primary antibodies were not used for immunocytochemical staining. After washing probes with PBS, the secondary antibodies were used for 1 h staining at room temperature. All the secondary antibodies were conjugated with either FITC or Texas Red. Cell nuclei were stained with 5 mM Hoechst 33258 (Sigma) for 30 min. After finally being washed, the slides were immersed in Fluoromount-G (Southern Biotechnology Association).

## 3. Results and Discussion

**Figure 4:**
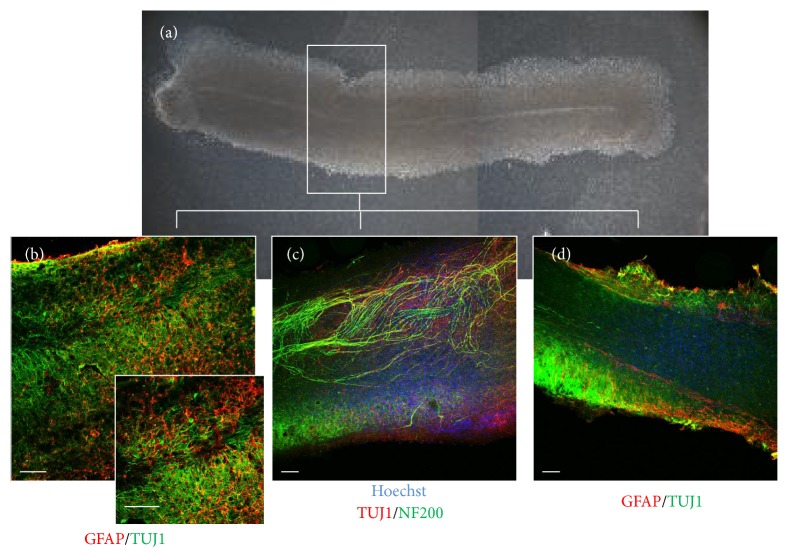
The intrinsic spinal cord axons forming a fiber tract. Two weeks after SCC preparation the anatomy of cultured slices was visualized by live imaging in light converted microscope (a) and by immunohistochemical analysis using neuronal and astrocytes markers ((b)–(d)). The scale bar is the equivalent of 200 *μ*m.

The method of establishing longitudinal spinal cord organotypic slice culture allows the observation of long fiber trajectory (Figures 4(b) and 4(c)), formation of new connections, and neurorepair processes occurring after the spinal cord injury. Thanks to the method of direct stem cell transplantation it is feasible to closely follow the fate of transplanted cells, their ability to differentiate (Figures 2(b) and 2(c)), and potential migration along axonal fibers. In addition, it allows the observation of the local inflammatory response to the graft caused by macrophage/microglia activation (visualized by staining with ED1) (Figure 2(d)) in respect of the stem cell source and the progress in their maturation process. Collecting both the slices and the transplanted stem cells after the experiment makes it possible to perform further molecular and biochemical analyses on the nucleic acids or protein level using different methods like PCR, ELISA, Western-blot, or immunohistochemistry.

Since most diseases of the spinal cord concern the adults, we have also established the longitudinal organotypic spinal cord slice culture from grown rats. Isolation of the core from adult individuals is, however, much more problematic. The fully myelinated spinal cord is very sensitive to any damage and cutting it with tissue chopper causes injury to about 60% of slices. The slice culture survives about 7 days maintaining proper parallel fiber tract architecture (see Supplementary Material available online at http://dx.doi.org/10.1155/2015/471216). While the neonatal slices display a constant high vitality level and tissue organization is preserved up to 4-5 weeks* in vitro*, the vitality of adult slice cultures decreases significantly upon the first 5 days of cultivation. At the same time the majority of cells in the neonatal slice culture were TUJ1-/NF200-positive, while NG2^+^ cells constituted a large fraction in the adult slice culture. The populations of astrocytes and microglia were comparable in the neonatal and adult organotypic spinal cord slice cultures (Supplementary Material).

The attempts to cultivate adolescent brain tissue for several weeks have been reported [34–36]. The oldest rodents used for organotypic tissue cultures of the hippocampus were 14–16 months old [37]. Although the published data confirm our observations that the fast processing cell death observed in the adult organotypic slice culture model is unfavorable to study long-term processes, for example, reinnervation, stem/progenitor cells differentiation, and maturation, but may serve as a potential model system to study neuroprotection, as suggested by others [38, 39].

**Figure 5:**
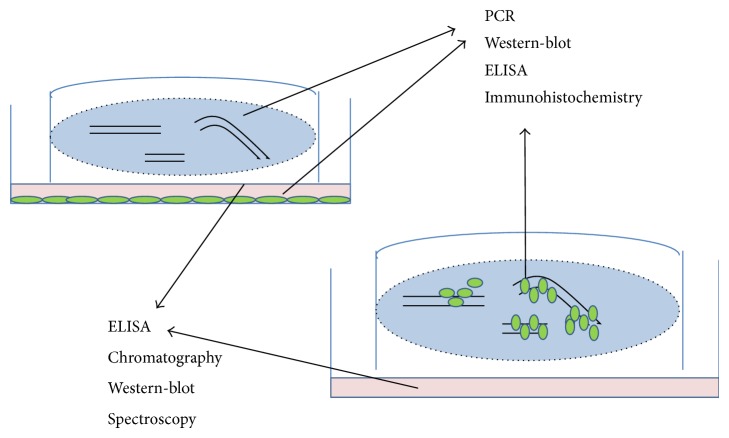
Possible applications of spinal cord organotypic slice culture. Spinal cord tissue after both direct and indirect cocultures with stem cells could be analyzed with immunohistochemical and molecular (PCR, Western-blot, ELISA, chromatography, and spectroscopy) methods.

Indirect cocultivation of the stem/progenitor cells along with SCC permit the analysis of the factors secreted into the medium by both stem cells and the spinal cord tissue and the determination of their influence on cell differentiation (Figure 3). The soluble factors could be identified and the paracrine mechanism in which they are engaged could be then determined by collecting culture media and subjecting them to analyses by means of ELISA, Western-blot, spectroscopy, or chromatography (Figure 5). Since elaborating a detailed protocol involved a great deal of work and was time-consuming, a list of potential difficulties, their probable causes, and the proposed solutions is enclosed in a separate table (Table 1).

**Figure 6:**
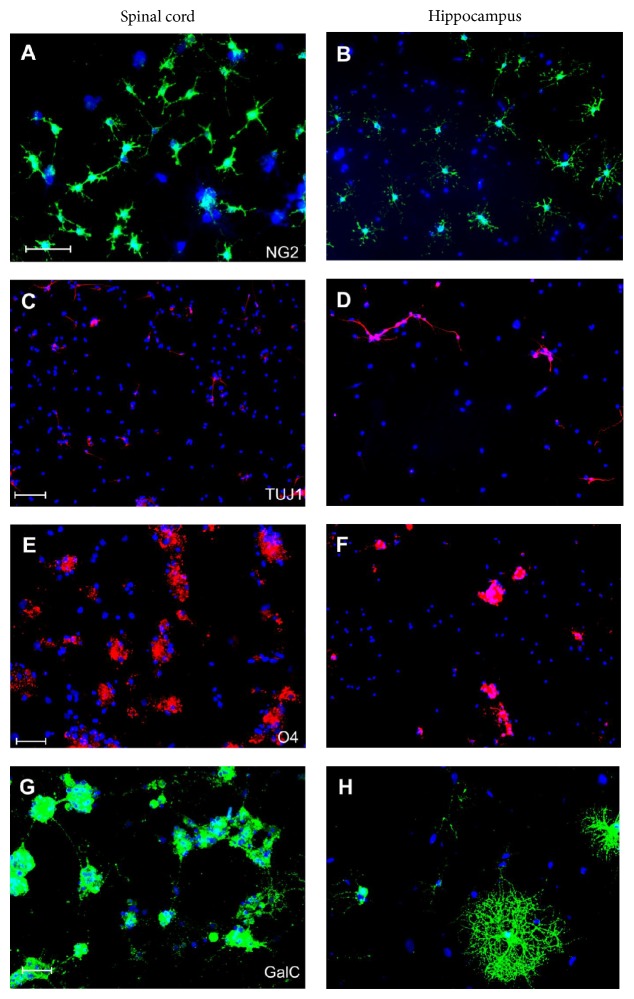
Local tissue (spinal cord versus hippocampus) significantly influences cell commitment, differentiation, and maturation, as observed during 7-day-long coculture experiments. The vicinity of hippocampal slices (right panel) promotes neuronal commitment and maturation (TUJ1) (D) and accelerates oligodendroglial differentiation into cells characterized by complex, multibranched morphology (GalC) (H). Conversely, in medium conditioned by the spinal cords slices (left panel), the progenitor and immature forms of oligodendrocytes are most abundant (NG2, O4) ((A), (E)), while GalC-positive cells begin to send out cell processes (G). Cell nuclei (blue) were stained with Hoechst 33258. Scale bar = 50 *μ*m.

The applications presented above are only the examples of how the SCC may be used for different research projects. However, there are many other techniques which could be used for SCC cultures, depending on the aim of the study (e.g., measuring enzyme activity, evaluating protective effects of various compounds, and determining signaling pathways in blocking experiments). Some new methods based on the application of organotypic slices are also being currently developed in our laboratory [40]. One of them concerned OPCs differentiation in vicinity of either spinal cord or hippocampal organotypic slices and could be an adequate example of the SCC application. Namely, to evaluate the influence of local tissue microenvironment on cell differentiation, neonatal rat OPCs were cocultured with organotypic slice culture derived from either the spinal cord or hippocampus (Figure 6) as described elsewhere [20]. First, we have observed that both direct and indirect cocultures with hippocampal slices promote neuronal commitment of a significant fraction of OPCs and acceleration of the oligodendroglial maturation [20, 30]. Just after 7 days of culturing, multibranched GalC-positive oligodendrocytes could be detected in a significant number (Figure 6). Conversely, the neurogenic effect was much less pronounced in the SCC-OPCs cocultures and cell differentiation proceeded much more slowly, when compared both to cocultures with hippocampal slices and to controls (OPCs alone). In the vicinity of slices derived from spinal cord the progenitor (NG2^+^) and immature (O4^+^) oligodendroglial cells predominated. The application of SCC in this study allowed us to prove that the local microenvironment has a significant impact on the cell commitment and differentiation. This observation contributes to the prediction of cell fate after their transplantation into spinal cord. In another project in which SCC were used, the astrocytic differentiation of OPCs was shown, which indicated their possible contribution to glial scar formation [21].

## 4. Conclusions

The presented method is the economical equivalent of the* in vivo* transplantation model used for studying spinal cord pathology. A trained user should be able to obtain 8–10 “workable” slices from a single rat spinal cord. Since the experience is the most important factor in getting satisfactory results, therefore the ideal situation is to assign 1 person to 2 persons to establish the right method in the laboratory and then to prepare slices for the whole research team.

## Supplementary Material

The adult slice culture survives about one week maintaining proper parallel fiber tract and proliferating ability. Most of the cells were NG2 positive, but also TUJ/NF200 expressing cells were present. The astrocytes and microglia populations, in the neonatal and adult organotypic spinal cord slice cultures were comparable.
Supplementary Figure 1: The longitudinal spinal cord slice culture derived from adult rats. 7 days after SCC preparation the anatomy of cultured slices was visualized by immunohistochemical analysis using neuronal (*β*- tubulin III), astrocytic (GFAP), oligodendrocyte (NG2) markers, as well as Ki67 – a marker of proliferating cells (A-D). The scale bar is the equivalent of 200 µm. Cell nuclei (blue) were visualized by Hoechst 33258.

## Figures and Tables

**Table 1:** Troubleshooting: the most frequent problems in preparing and maintaining the organotypic longitudinal spinal cord slice culture, their probable reasons, and their proposed solutions.

Problems	Possible reason	Solutions
Spinal cord destroyed during chopping	The tissue is too soft	All the procedures must be done on ice and the chopper table should be chilled before cutting
	The blade cuts too fast and tears the tissue up	The blade speed should be approximately adjusted to make a single cut every single second
	The blade does not stick closely to the chopper table	Make sure that the blade tightly sticks to the chopper table

Aberrant axonal projections	The spinal cord is not placed in a parallel-to-the-blade fashion	Before cutting check whether the spinal cord is set parallel to the blade.

Slices die after 1 week	Incorrect pH of the medium	pH must be adjusted to 7.2 each time the medium is changed
	The precutting procedure takes too much time	The whole procedure should not take more than 90 min (from decapitating animals up to placing the slices onto the membrane)

Transplanted cells die after short time	The density of the transplanted cells is too high	Try different amount of cells
	The cells are not evenly suspended	Before transplantation mix cells in eppendorf with pipette gently but precisely

Slices and cells detach from the membrane during fixation	PFA was old or too cold	After preparing PFA solution do not freeze the preused doses and before fixation heat PFA up to 37°C

Slices are not evenly stained	The permeabilization is too weak or too short	Add Triton-X 0.2% to the primary antibody solution
	The slice was not completely covered with liquid	Using a brush or tips let the slice gently sink to the bottom of the well; it should stay there during entire staining procedure

The slice structure is destroyed during slide closing	The coverslip is too heavy and crushes are rich in lipids structure	Add a double portion of mounting medium and wait a while until it dries a little before applying coverslips

## Abbreviations

CMFDA: 5-Cloromethylfluorescein diacetate
CNS: Central nervous system
HUCB-NSC: Neural stem cells derived from human umbilical cord blood
OPCs: Oligodendrocyte progenitor cells
SCC: Spinal cord organotypic slice culture
SF: Serum free
PFA: Paraformaldehyde.
