# Supplementary figures and images for: The Organotypic Longitudinal Spinal Cord Slice Culture for Stem Cell Study
Source: Stem Cells Int. 2015 Jan 31;2015:471216. doi: 10.1155/2015/471216 (PMC4329758; doi:10.1155/2015/471216)

**Ki67/ GFAP**

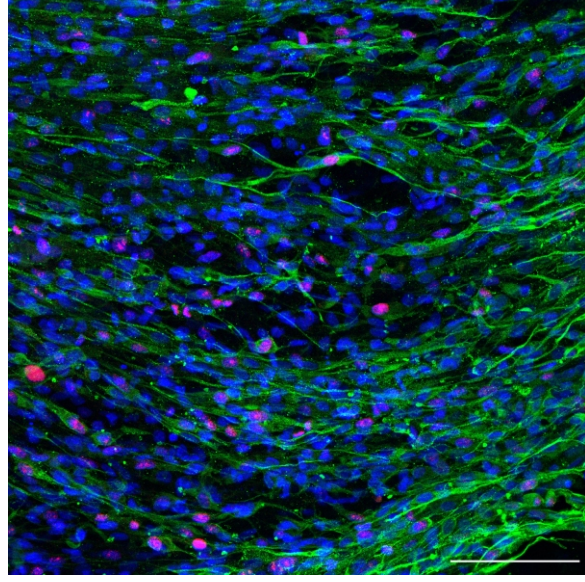

**III tubulin**

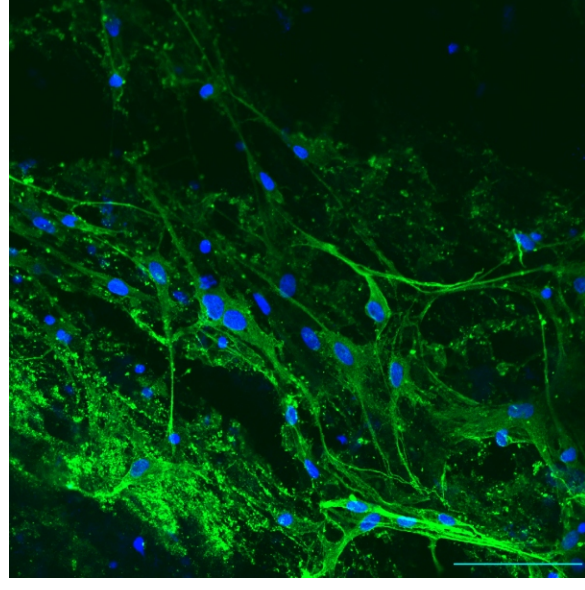

**NG2**

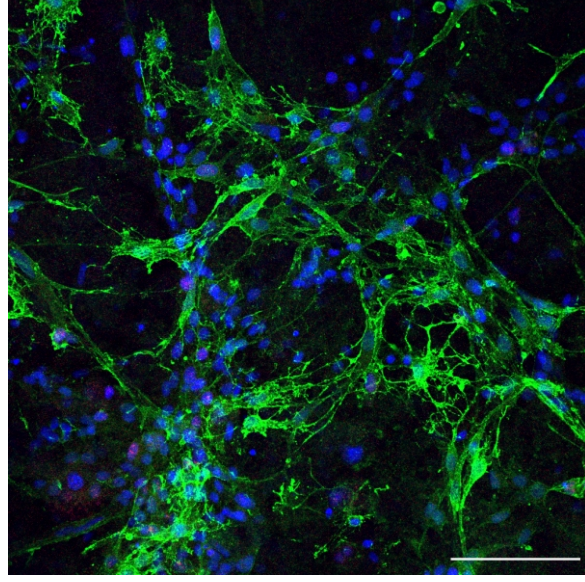

**GFAP/ III tubulin**

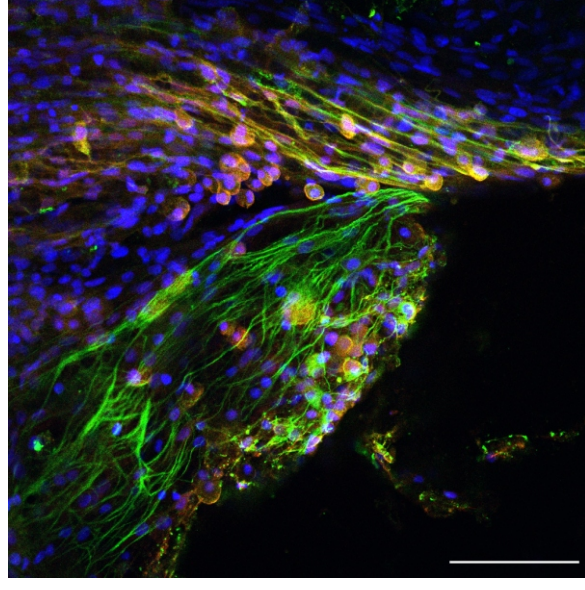

Supplement: Supplementary file 1 — The adult slice culture survives about one week maintaining proper parallel fiber tract and proliferating ability. Most of the cells were NG2 positive, but also TUJ/NF200 expressing cells were present. The astrocytes and microglia populations, in the neonatal and adult organotypic spinal cord slice cultures were comparable. Supplementary Figure 1: The longitudinal spinal cord slice culture derived from adult rats. 7 days after SCC preparation the anatomy of cultured slices was visualized by immunohistochemical analysis using neuronal (β- tubulin III), astrocytic (GFAP), oligodendrocyte (NG2) markers, as well as Ki67 – a marker of proliferating cells (A-D). The scale bar is the equivalent of 200 µm. Cell nuclei (blue) were visualized by Hoechst 33258. [file 471216.f1.pdf]
